# An evaluation of reliability in measurement of native femoral torsion on CT imaging

**DOI:** 10.1007/s00590-025-04637-2

**Published:** 2025-12-24

**Authors:** M. Kareem Shaath, David Ahn, Joseph A. Ippolito, Jonathan Lee, Joseph Galloway, Michael S. Sirkin, Mark C. Reilly, Mark R. Adams

**Affiliations:** 1 Orlando Health Jewett Orthopaedic Institute, Orlando, FL USA; 2 Rutgers New Jersey Medical School, Newark, NJ USA; 3 Englewood Hospital and Medical Center, Englewood, NJ USA; 4 University of South Florida, Tampa, FL USA

**Keywords:** Femoral torsion, CT scanogram, Interobserver reliability, Intraobserver reliability

## Abstract

**Background:**

This study assesses intra- and interobserver reliability in measuring femoral torsion on repeated computed tomography (CT) scanograms and explores variability across training levels and patient characteristics.

**Methods:**

Patients from 2001 to 2017 with femur fractures who underwent multiple CT scanograms were included. Measurements were performed by an orthopaedic traumatologist, two orthopaedic residents (PGY-5 and PGY-3), and a musculoskeletal radiologist. Intraobserver reliability was assessed using intraclass correlation coefficients (ICCs), and interobserver reliability using repeated measures ANOVA. Correlations between measurement variability and body mass index (BMI), age, height, gender, and laterality were evaluated using Pearson’s coefficient.

**Results:**

Twenty-nine patients with unilateral uninjured femurs were analyzed. Interobserver analysis revealed significant differences in femoral torsion (*p* = 0.006) and length (*p* = 0.019). Intraobserver reliability showed strong agreement in femoral torsion (ICC 0.620–0.950) and length (ICC 0.992–0.999), but moderate to poor agreement in femoral neck axis (ICC 0.394–0.627) and posterior condylar axis (ICC 0.561–0.665). Femoral length measurements were the most consistent across all reviewers. Higher BMI correlated with increased variability in femoral torsion measurements (*r* = 0.378; *p* = 0.048). No significant correlation was found for age (*p* = 0.110), height (*p* = 0.363), gender (*p* = 0.610), or laterality (*p* = 0.830).

**Conclusion:**

These findings highlight the reliability of CT scanograms to assess femoral length and torsion when done by the same physician. Although moderate to poor agreement in femoral neck axis and posterior femoral condyle axis, overall femoral torsion was found to be highly reliable. Reliable measurement of native femoral torsion in patients with a higher BMI may be difficult. To ensure reliable measures of femoral torsion, the same clinician should evaluate all pertinent studies for an individual patient.

**Level of evidence:**

Level III Retrospective cohort study.

## **Introduction**

For certain femoral shaft fracture patterns, restoring the length and rotation of the limb during a closed intramedullary nailing can be challenging. As malrotation can negatively affect the patellofemoral joint and the patient’s overall function, accuracy in femoral torsion is one of the goals of this operation [1, 2]. Although differences in the rotation between a patient’s right and left native femurs can exist [3], the goal for surgery is for rotational symmetry. As clinical examination is not sufficient to accurately evaluate the rotation of the femur, a postoperative computed tomography (CT) scanogram is used to verify the rotation of the femur postoperatively in select cases [4]. Unlike standard CT scans, a scanogram acquires images at specific anatomical landmarks—typically the hip, knee, and ankle—allowing for precise measurement of the length of the femur and comparison between the injured and contralateral side.

The goal of the surgery is to have the injured femur within 10 to 15 degrees of the rotation of the contralateral femur [5]. If the patient’s rotation is outside of these acceptable limits, revision surgery is often indicated, as altered patellofemoral joint mechanics, difficulty with climbing stairs, anterior knee pain, and overall suboptimal patient outcomes are often associated with rotational misalignment [6, 7]. However, patients and surgeons are reluctant to undergo revision surgery. The surgeon needs confidence in the reliability of the CT scanogram to make this decision. Furthermore, after a revision surgery is performed, a second CT scanogram can be used to confirm the rotational correction [8]. When two tests are performed, there is the potential for the intraobserver and interobserver variability between the two studies to become problematic [4]. 

To ascertain whether rotational measurements can be made consistently, we examined the intact femur of patients that had two CT scanograms. Our hypothesis was that there is no difference in overall femoral torsion and length on repeat CT scanogram imaging of a patient’s intact femur. We also aimed to assess of the reliability of measurements of femoral torsion across different levels and backgrounds of training and to identify factors that may contribute to variability in measuring overall femoral torsion.

## **Methods**

After Institutional Review Board approval, patients were retrospectively identified from our institution’s electronic medical record database from 2001 to 2017 who underwent treatment of either a femoral shaft fracture with an intramedullary implant (CPT Code 27506) or an intertrochanteric, pertrochanteric, or subtrochanteric femoral fracture treated with an intramedullary implant (CPT Code 27245). Inclusion criteria included patients with multiple CT scanograms of an uninjured femur with a corresponding axis measurement performed and dictated by a musculoskeletal radiologist. Exclusion criteria included patients under the age of 18, incomplete chart data, and bilateral femur fractures. A total of 29 eligible patients were included and analyzed: patient demographic details, including age, gender, BMI, and laterality were collected and are summarized in Table 1. All imaging studies and operative records were exclusively obtained from our institution’s Level 1 Trauma Center database. The primary outcome of this study was to assess intra- and interobserver reliability of femoral torsion measurements on repeat CT scans. Secondary outcomes included evaluation of intra- and interobserver reliability for femoral neck axis, posterior femoral condylar axis, and femoral length, as well as correlations between measurement variability and patient characteristics including BMI, age, height, gender, and laterality.

**Table 1:** Variability of measurements (Initial vs. Repeat CT)

Measure	Mean variability ± SD	*p*-value*	Partial η²
Femoral torsion			
Orthopaedic Traumatologist	3.1 ± 2.0 degrees	0.006	0.16
Orthopaedic Resident (PGY-5)	4.1 ± 3.6 degrees		
Orthopaedic Resident (PGY-3)	2.2 ± 2.0 degrees		
Radiologist	5.9 ± 6.6 degrees		
*Femoral Neck Axis*			
Orthopaedic Traumatologist	8.3 ± 5.9 degrees	0.291	0.12
Orthopaedic Resident (PGY-5)	7.2 ± 5.6 degrees		
Orthopaedic Resident (PGY-3)	9.2 ± 5.8 degrees		
Radiologist	8.8 ± 6.4 degrees		
*Posterior Femoral Condyle Axis*			
Orthopaedic Traumatologist	10.3 ± 6.2 degrees	0.65	0.08
Orthopaedic Resident (PGY-5)	9.4 ± 6.2 degrees		
Orthopaedic Resident (PGY-3)	10.9 ± 6.7 degrees		
Radiologist	10.8 ± 7.0 degrees		
*Femoral Length*			
Orthopaedic Traumatologist	1.3 ± 1.4 mm	0.019	0.78
Orthopaedic Resident (PGY-5)	3.0 ± 3.3 mm		
Orthopaedic Resident (PGY-3)	1.5 ± 1.1 mm		
Radiologist	2.3 ± 2.3 mm		

*p-value were calculated based on repeated measures ANOVA

**Fig. 1:**
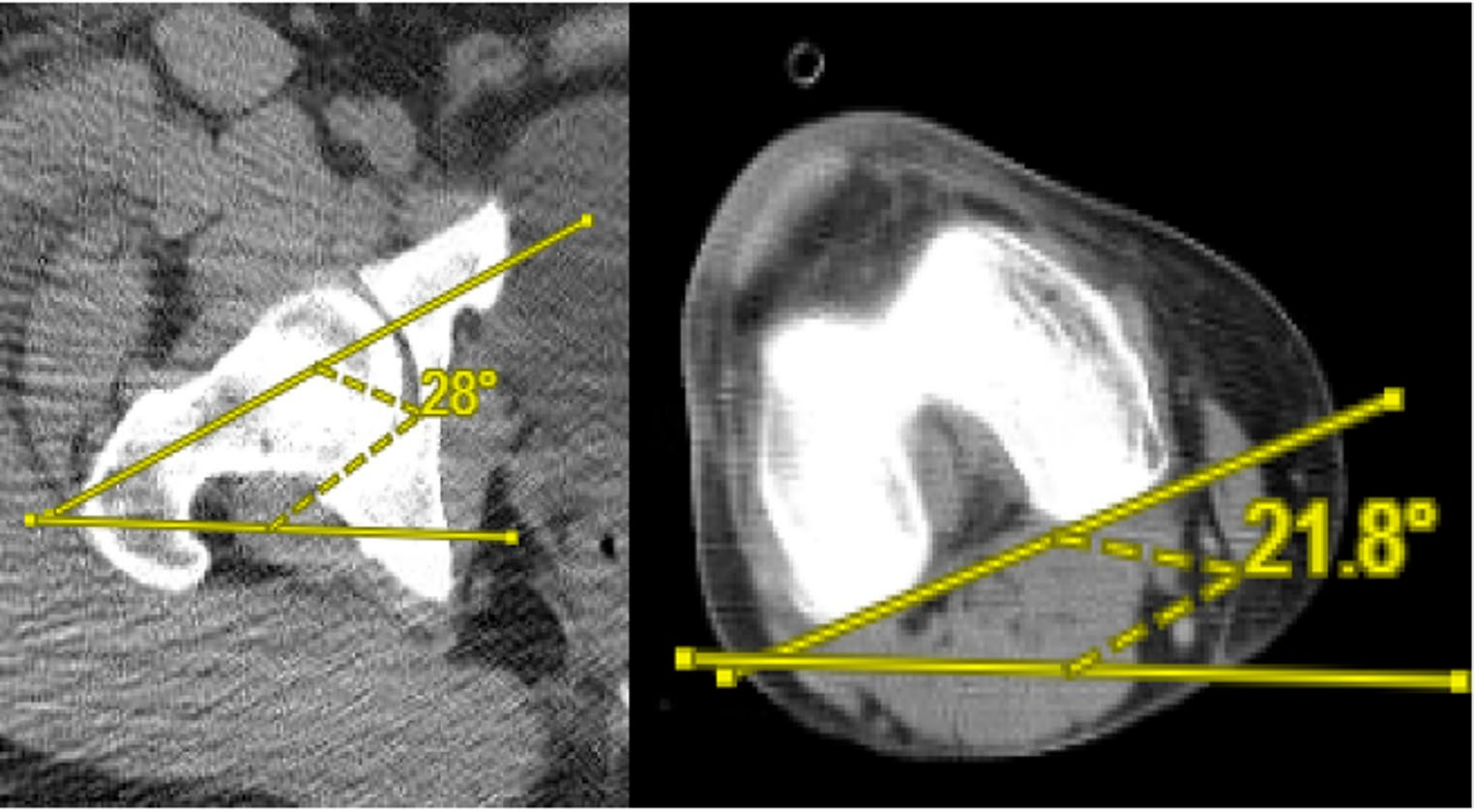
Description of measure of femoral torsion. (**A**; Left Panel) Measure of femoral neck axis is performed by drawing a line through the axis of the femoral neck and referencing to a horizontal line. (**B**; Right Panel) Measure of posterior femoral condyle axis is performed by drawing a line tangential to the posterior aspect of the femoral condyles and again referencing to the horizontal. To calculate femoral torsion, the axis at the posterior femoral condyles is subtracted from axis at the femoral neck

The measurements of the femoral neck axis, posterior femoral condylar axis, femoral torsion, and femoral length of the uninjured femur were conducted by a team consisting of an orthopaedic traumatologist, a PGY-5 orthopaedic resident, a PGY-3 orthopaedic resident, and a musculoskeletal radiologist. CT scanograms were blinded and randomized prior to measurement. Each of the four reviewers independently evaluated the randomized, blinded CT scanograms were measured, and each reviewer obtained the femoral neck axis, posterior condylar axis, femoral torsion, and femoral length once per scan. The imaging data were analyzed using specialized software (Centricity™ PACS-IW, GE Healthcare IT, Little Chalfont, UK). All measurements were completed as described by Jeanmart et al. and modified by Dugdale et al. (Fig. 1) [8, 9]. 

For interobserver reliability, repeated measures ANOVA was utilized. For intraobserver reliability, intraclass coefficients (ICC) were generated utilizing a two-way random effects model for measurements of femoral neck axis, posterior femoral condyle axis, femoral torsion, and femoral length. Additionally, utilizing Pearson’s correlation coefficient, the following variables were assessed as potential predictors for variability: BMI, age, height, gender, and laterality. Significant agreement was considered to correspond to a *p* value of < 0.05. Statistical analysis was performed using GraphPad Prism version 7.00 for MAC, (GraphPad Software, La Jolla, CA, USA) and SPSS data analysis software (version 1.0.0. 1347 for MAC, IBM Corp. Armonk, NY, USA).

## **Results**

Twenty-nine patients were retrospectively identified who had multiple CT scanograms of an uninjured femur. There were 25 males and 4 females included for analysis. Mean age was 30.9 ± 10.9 years, mean BMI was 27.4 ± 4.8, mean height was 176.8 ± 8.4 cm, and mean weight was 85.8 ± 17.1 kg. A total of 15 left femurs and 14 right femurs were included for final analysis.

Overall, mean measurements among all reviewers between the first and second CT scanograms were 8.7 ± 9.4° for femoral torsion, 12.3 ± 10.7° for femoral neck axis, −3.6 ± 13.2° for posterior femoral condyle axis, and 480.5 ± 35.2 mm for femoral length. On repeated measures ANOVA for interobserver reliability, the four types of observers (orthopaedic traumatologist, PGY-5 orthopedic resident, PGY-3 orthopedic resident, and musculoskeletal radiologist) differed in femoral torsion measurements (F(1.50, 40.36) = 5.05, *p* = 0.006, partial η^2^ = 0.16), and in femoral length measurements (F(3, 25) = 29.95, *p* = 0.019, partial η^2^ = 0.78), representing medium-large and very large effect sizes, respectively. No statistically significant differences were observed for the femoral neck axis (F(3, 25) = 1.12, *p* = 0.29, partial η^2^ = 0.12) or posterior femoral condylar axis (F(3, 25) = 0.68, *p* = 0.65, partial η^2^ = 0.08), which correspond to small-to-moderate effects (Table 1).

Strong agreement was found between first and second measurements of femoral torsion (0.620–0.950) and femoral length (0.992–0.999). Moderate to poor agreement was found between first and second measurements of femoral neck axis and posterior femoral condyle axis (0.394–0.627 and 0.561–0.665, respectively) (Table 2).

**Table 2:** Intraobserver agreement between first and second CT measurements, measured by intraclass coefficient (ICC)^a^

Rater	Femoral neck axis	Posterior femoral condyle axis	Femoral torsion	Femoral length
Orthopaedic Traumatologist	0.622	0.592	0.924	0.999
Orthopaedic Resident (PGY-5)	0.627	0.665	0.819	0.992
Orthopaedic Resident (PGY-3)	0.557	0.572	0.950	0.999
Radiologist	0.394	0.561	0.620	0.996

^a^ICC was calculated for each reviewer comparing two CT scans of the same unaffected femur for each patient. All measurements were significantly correlated (*p* < 0.05)

BMI was positively correlated with increased variability in measure of femoral torsion (*r* = 0.378; *p* = 0.048), while age (*p* = 0.110), height (*p* = 0.363), gender (*p* = 0.610), and laterality (*p* = 0.830) were not (Table 3).

**Table 3:** Predictors of variability in measures of femoral torsion

Variable	Pearson coefficient	*p*-value
BMI	0.378	*0.048*
Age	0.303	0.110
Height	0.179	0.363
Gender	0.099	0.610
Laterality	0.042	0.830

The italics are there to demonstrate that the *p*-valuee is less than 0.05

## **Discussion**

Femoral malrotation is a known complication following intramedullary (IM) nailing of femoral shaft fractures, with its incidence well-documented [4, 10, 11]. Clinical sequelae can include gait disturbances, altered patellofemoral mechanics, and difficulties with stairs, underscoring the importance of accurate rotational alignment [4, 6, 7, 10–13]. In this study, 29 patients underwent multiple CT scanograms of an uninjured femur, revealing strong intraobserver agreement for femoral torsion (ICC 0.620–0.950) and femoral length (ICC 0.992–0.999). We also found moderate to poor agreement for the femoral neck axis and posterior condylar axis (0.394–0.627 and 0.561–0.665, respectively), suggesting variability in the patient positioning between the first and second CT scan acquisitions. Despite these axis-level fluctuations, overall femoral torsion remained stable, suggesting that regardless of the variability in patient positioning, CT scans are reliable in determining a patient’s femoral torsional profile. Additionally, interobserver variability was significant for femoral torsion (*p* = 0.006) and femoral length (*p* = 0.019), indicating that when a repeat CT scanogram is performed, the same observer should ideally take both measurements. Moreover, we noted a positive correlation between higher BMI and increased torsion variability, aligning with reports that landmarks may become obscured in heavier patients [14–18].

Our findings reinforce the value of CT scanograms in verifying femoral torsion and length [4, 12]. Postoperative difference of more than 10–15° in torsion may prompt revision [5], and a reliable imaging modality is therefore critical for decision-making. Notably, most prior research on interobserver and intraobserver reliability evaluated the same study multiple times [4, 13]. By contrast, the use of two separate CT scanograms in this investigation underscores that, under real‐world conditions, repeated imaging—if assessed by a consistent observer—can produce reproducible torsion and length measurements, thus helping address doubts about the need for revision surgery [2, 6, 7]. 

Although these data lend support to the consistency of repeat CT scanograms, they do not provide absolute validation. A future cadaveric study comparing CT torsion measurements to direct anatomic observations would confirm how closely scanogram results reflect true femoral geometry [8, 9]. Another limitation is our limited number of observers, selected to represent typical clinical roles (orthopaedic traumatologist, musculoskeletal radiologist, and orthopaedic residents). Increasing the pool of graders in a larger, prospective study would help refine and validate interobserver reliability further. Finally, although we specifically evaluated uninjured femurs, in real-world clinical practice repeated CT scanograms often focus on injured femurs—especially to reassess alignment before or after malrotation revision. By isolating intact femurs, our study provides foundational evidence of reliable intraobserver measurements without the added complexities of hardware or fracture healing. Nonetheless, because surgical correction commonly aims to restore the injured femur within 10–15° of the contralateral side, demonstrating high intraobserver agreement in uninjured femurs helps reinforce confidence that a repeat scan—when read by the same clinician—will not exceed this acceptable threshold.

Clinically, these results suggest that when a CT scan indicates possible malrotation, obtaining a repeat scan and having the same clinician perform the measurement may increase confidence in the assessment. This is particularly relevant for surgeons and patients deliberating a revision procedure, given the potential physical and financial implications. In patients with elevated BMI, where anatomic landmarks may be less distinct, adherence to standardized positioning protocols or use of software-based measurement tools may further improve consistency. Future research should aim to include larger, prospective cohorts and a greater number of observers to confirm these findings and refine reliability thresholds for routine clinical use.

While the sample size and limited number of reviewers constrain the statistical power of this study, the results nonetheless provide valuable preliminary insight into the reproducibility of femoral torsion measurements using repeat CT scans. These findings support the feasibility and potential reliability of this technique in practice, particularly when assessments are performed by the same observer. Larger, multicenter investigations are warranted to validate these trends and determine their generalizability across broader patient populations and clinical settings.

## Data Availability

No datasets were generated or analysed during the current study.

## References

[1] Jaarsma RL, Ongkiehong BF, Grüneberg C, Verdonschot N, Duysens J, van Kampen A (2004) Compensation for rotational malalignment after intramedullary nailing for femoral shaft fractures. An analysis by plantar pressure measurements during gait. Injury 35(12):1270–1278. 10.1016/j.injury.2004.01.016 15561117 10.1016/j.injury.2004.01.016

[2] Gugenheim JJ, Probe RA, Brinker MR (2004) The effects of femoral shaft malrotation on lower extremity anatomy. J Orthop Trauma 18(10):658–664. 10.1097/00005131-200411000-00002 15507818 10.1097/00005131-200411000-00002

[3] Reikerås O, Høiseth A, Reigstad A, Fönstelien E (1982) Femoral neck angles: a specimen study with special regard to bilateral differences. Acta Orthop Scand 53(5):775–779. 10.3109/17453678208992291 7136588 10.3109/17453678208992291

[4] Jaarsma RL, Pakvis DF, Verdonschot N, Biert J, van Kampen A (2004) Rotational malalignment after intramedullary nailing of femoral fractures. J Orthop Trauma 18(7):403–409. 10.1097/00005131-200408000-00002 15289684 10.1097/00005131-200408000-00002

[5] Bråten M, Terjesen T, Rossvoll I (1993) Torsional deformity after intramedullary nailing of femoral shaft fractures. Measurement of anteversion angles in 110 patients. J Bone Joint Surg Br 75(5):799–803. 10.1302/0301-620x.75b5.8376444 8376444 10.1302/0301-620X.75B5.8376444

[6] Yildirim AO, Aksahin E, Sakman B, Kati YA, Akti S, Dogan O et al (2013) The effect of rotational deformity on patellofemoral parameters following the treatment of femoral shaft fracture. Arch Orthop Trauma Surg 133(5):641–648. 10.1007/s00402-013-1705-x 23443529 10.1007/s00402-013-1705-x

[7] Karaman O, Ayhan E, Kesmezacar H, Seker A, Unlu MC, Aydingoz O (2014) Rotational malalignment after closed intramedullary nailing of femoral shaft fractures and its influence on daily life. Eur J Orthop Surg Traumatol 24(7):1243–1247. 10.1007/s00590-013-1289-8 23934503 10.1007/s00590-013-1289-8

[8] Jeanmart L, Baert AL, Wackenheim A (1983) Computer tomography of neck, chest, spine, and limbs. Atlas of pathological computer tomography. Springer, Cham

[9] Dugdale TW, Degnan GG, Turen CH (1992) The use of computed tomographic scan to assess femoral malrotation after intramedullary nailing. A case report. Clin Orthop Relat Res. 279:258–263 1600664

[10] Wolinsky P, Tejwani N, Richmond JH, Koval KJ, Egol K, Stephen DJ (2002) Controversies in intramedullary nailing of femoral shaft fractures. Instr Course Lect 51:291–303 12064115

[11] Yang KH, Han DY, Jahng JS, Shin DE, Park JH (1998) Prevention of malrotation deformity in femoral shaft fracture. J Orthop Trauma 12(8):558–562. 10.1097/00005131-199811000-00005 9840789 10.1097/00005131-199811000-00005

[12] Lindsey JD, Krieg JC (2011) Femoral malrotation following intramedullary nail fixation. J Am Acad Orthop Surg 19(1):17–26. 10.5435/00124635-201101000-00003 21205764 10.5435/00124635-201101000-00003

[13] Yoon RS, Koerner JD, Patel NM, Sirkin MS, Reilly MC, Liporace FA (2013) Impact of specialty and level of training on CT measurement of femoral version: an interobserver agreement analysis. J Orthop Traumatol 14(4):277–281. 10.1007/s10195-013-0263-x 23989857 10.1007/s10195-013-0263-x PMC3828493

[14] Le NT, Robinson J, Lewis SJ (2015) Obese patients and radiography literature: what do we know about a big issue? J Med Radiat Sci 62(2):132–141. 10.1002/jmrs.105 26229678 10.1002/jmrs.105 PMC4462985

[15] Uppot RN (2018) Technical challenges of imaging & image-guided interventions in obese patients. Br J Radiol 91(1089):20170931. 10.1259/bjr.20170931 29869898 10.1259/bjr.20170931 PMC6223172

[16] Feldmane I, Gampp C, Hausmann D, Mavridis S, Euler A, Hefermehl LJ et al (2023) Evaluation of image quality of overweight and obese patients in CT using high data rate detectors. Vivo 37(3):1186–1191. 10.21873/invivo.13194 10.21873/invivo.13194 PMC10188035 37103075

[17] Ghanem MA, Kazim NA, Elgazzar AH (2011) Impact of obesity on nuclear medicine imaging. J Nucl Med Technol 39(1):40. 10.2967/jnmt.110.078881 21321247 10.2967/jnmt.110.078881

[18] Fursevich DM, LiMarzi GM, O’Dell MC, Hernandez MA, Sensakovic WF (2016) Bariatric CT imaging: challenges and solutions. Radiographics 36(4):1076–1086. 10.1148/rg.2016150198 27232505 10.1148/rg.2016150198

